# Systems-Wide Prediction of Enzyme Promiscuity Reveals a New Underground Alternative Route for Pyridoxal 5’-Phosphate Production in *E*. *coli*

**DOI:** 10.1371/journal.pcbi.1004705

**Published:** 2016-01-28

**Authors:** Matthew A. Oberhardt, Raphy Zarecki, Leah Reshef, Fangfang Xia, Miquel Duran-Frigola, Rachel Schreiber, Christopher S. Henry, Nir Ben-Tal, Daniel J. Dwyer, Uri Gophna, Eytan Ruppin

**Affiliations:** 1 School of Computer Sciences and Sackler School of Medicine, Tel Aviv University, Tel Aviv, Israel; 2 Department of Molecular Microbiology and Biotechnology, Faculty of Life Sciences, Tel Aviv University, Tel Aviv, Israel; 3 Center for Bioinformatics and Computational Biology, Department of Computer Science, University of Maryland, College Park, Maryland, United States of America; 4 Mathematics and Computer Science Division, Argonne National Laboratory, Argonne, Illinois, United States of America; 5 Joint IRB-BSC-CRG Program in Computational Biology, Institute for Research in Biomedicine (IRB Barcelona), Barcelona, Spain; 6 Department of Biochemistry and Molecular Biology, George S. Wise Faculty of Life Sciences, Tel Aviv University, Tel Aviv, Israel; 7 Department of Cell Biology and Molecular Genetics, Institute for Physical Science and Technology, Department of Bioengineering, Maryland Pathogen Research Institute, University of Maryland, College Park, Maryland, United States of America; University of California San Diego, UNITED STATES

## Abstract

Recent insights suggest that non-specific and/or promiscuous enzymes are common and active across life. Understanding the role of such enzymes is an important open question in biology. Here we develop a genome-wide method, PROPER, that uses a permissive PSI-BLAST approach to predict promiscuous activities of metabolic genes. Enzyme promiscuity is typically studied experimentally using multicopy suppression, in which over-expression of a promiscuous ‘replacer’ gene rescues lethality caused by inactivation of a ‘target’ gene. We use PROPER to predict multicopy suppression in *Escherichia coli*, achieving highly significant overlap with published cases (hypergeometric p = 4.4e-13). We then validate three novel predicted target-replacer gene pairs in new multicopy suppression experiments. We next go beyond PROPER and develop a network-based approach, GEM-PROPER, that integrates PROPER with genome-scale metabolic modeling to predict promiscuous replacements via alternative metabolic pathways. GEM-PROPER predicts a new indirect replacer (*thiG*) for an essential enzyme (*pdxB*) in production of pyridoxal 5’-phosphate (the active form of Vitamin B_6_), which we validate experimentally via multicopy suppression. We perform a structural analysis of *thiG* to determine its potential promiscuous active site, which we validate experimentally by inactivating the pertaining residues and showing a loss of replacer activity. Thus, this study is a successful example where a computational investigation leads to a network-based identification of an indirect promiscuous replacement of a key metabolic enzyme, which would have been extremely difficult to identify directly.

## Author Summary

Many enzymes can perform secondary functions at low affinities or rates, but such ‘promiscuous’ functions have never been predicted on a genome-wide scale. Here, we present the first genome-wide method to predict promiscuous functions of metabolic genes, which we apply to *E*. *coli*. Notably, we predict and validate several new cases where a ‘replacer’ gene can compensate for the loss of an essential ‘target’ gene through a promiscuous activity. Next, we couple our method with a genome-scale metabolic model, in order to search for ‘replacer’ genes that compensate for essential ‘target’ genes by metabolically bypassing them. We use this network-augmented approach to uncover a novel promiscuous pathway for the production of pyridoxal 5’-phosphate (the active form of Vitamin B_6_) in *E*. *coli*. This study represents an important step in understanding promiscuous functions in bacteria, and is a prime example of a systems-level analysis leading to new biological insight.

## Introduction

Enzymes have traditionally been associated with discrete activities [1]. Clear-cut annotations populate well-known enzyme databases such as KEGG and Uniprot, and foster an implicit assumption that gene activities are specific. However, recent advancements in our understanding of evolution and enzyme activity have cast this view into question, and suggest instead that non-optimized and/or promiscuously active enzymes are frequent and active across life [2]. ‘Generalist’ enzymes (i.e., those carrying more than one function) have been shown to be abundant, to play different biological roles than ‘specialist’ enzymes, and to behave differently than specialist enzymes during switches in media conditions [3]. Promiscuous enzyme activities (i.e., those that are only active with low affinities/activities) have been shown to play adaptive biological roles (e.g., [4] and [5]), and to often arise through neutral mutations that are not detrimental to the primary enzymatic activity [6]. In particular, antibiotic resistance may emerge initially through the action of over-expressed, promiscuous genes [7, 8].

Because of recent conceptual and modeling advances, there is significant interest in being able to predict and exploit enzyme promiscuity [9–12]. Tools have been developed that mine molecular signatures or three-dimensional catalytic domains of enzymes to predict whether enzymes are promiscuous [13–15], and these methods have been used, e.g., to design likely retrosynthetic pathways [9, 11]. While these methods achieve important goals, they have not been aimed at evaluating the functional implications of *existing* promiscuous activities on a genome-wide, network scale. A notable recent effort did probe the global relevance of promiscuous enzyme activities, but the study used ‘as is’ data collected from enzyme databases, which, while having the advantage of being fully experimentally verified, include many non-biologically-relevant instances and exclude many biologically relevant ones [16]. Another recent study used knowledge gaps identified in a genome-scale metabolic model of *E*. *coli* to identify candidate isozymes, which display substrate promiscuity [17]. In that study, a ‘target’ enzyme that is found to be nonessential *in vitro* despite being essential *in silico* is knocked out, after which potential isozymes that are found to be upregulated are cumulatively knocked out until the cell can no longer survive. This method thus identifies enzymes with a secondary isozyme function that, when the cell overexpresses them, can compensate for the loss of the ‘target’. Interestingly, in one case they consider, the target is in fact essential, and adaptive evolution is required for the potential isozyme to be expressed enough to compensate for its loss. Searching for many more such low activity promiscuous functions in an unsupervised, genome-wide way is the focus of this present study.

To accomplish this, we utilize an unsupervised PSI-BLAST based method for assessing potential secondary functions of genes in *E*. *coli*. We predict promiscuous ‘replacer’ functions that may compensate for primary ‘target’ functions in other genes if they are altered or lost, and compare these predictions to known cases in which an over-expressed gene can take on a secondary role, as shown in an assay generally referred to as multicopy suppression [18]. Multicopy suppression reveals ‘replacer’ genes whose over-expression suppresses the lethality caused by knocking out conditionally essential ‘target’ genes. It is thus an elegant assay for uncovering promiscuous gene functions. We find that our method for predicting promiscuous functions successfully recapitulates known multicopy suppression events [8, 19] and predicts new ones, several of which we validate *in vitro*.

We next develop a genome-scale metabolic modeling (**GEM**) based approach to predict and experimentally test cases of multicopy suppression in which functional promiscuous replacement happens ‘indirectly’ through a bypassing pathway or reaction, rather than by directly replacing the function of the target gene. For this, we focus on the gene target *pdxB*, for which multiple promiscuous replacers have been reported in the past [8]. PdxB is a key enzyme for producing pyridoxal 5’-phosphate (the active form of Vitamin B6), an essential nutrient in *E*. *coli*. Our experiment proceeded in 4 steps: First, we predicted that the gene *thiG* harbors a promiscuous activity that metabolically bypasses *pdxB*. Second, we validated this prediction with a new *in vitro* individual multicopy suppression experiment. Third, we performed a detailed structural analysis of ThiG and hypothesized which residues perform the promiscuous function. And Fourth, we mutated this active site and confirmed loss of *pdxB* replacement activity. Thus, we begin at the level of a genome-wide screen for promiscuous functions, and proceed to identify and validate a promiscuous pathway for production of an essential nutrient in *E*. *coli*, which could have important functional implications for this model organism.

## Results

### Developing an enzyme PROmiscuity PrEdictoR (PROPER)

**Fig 1:**
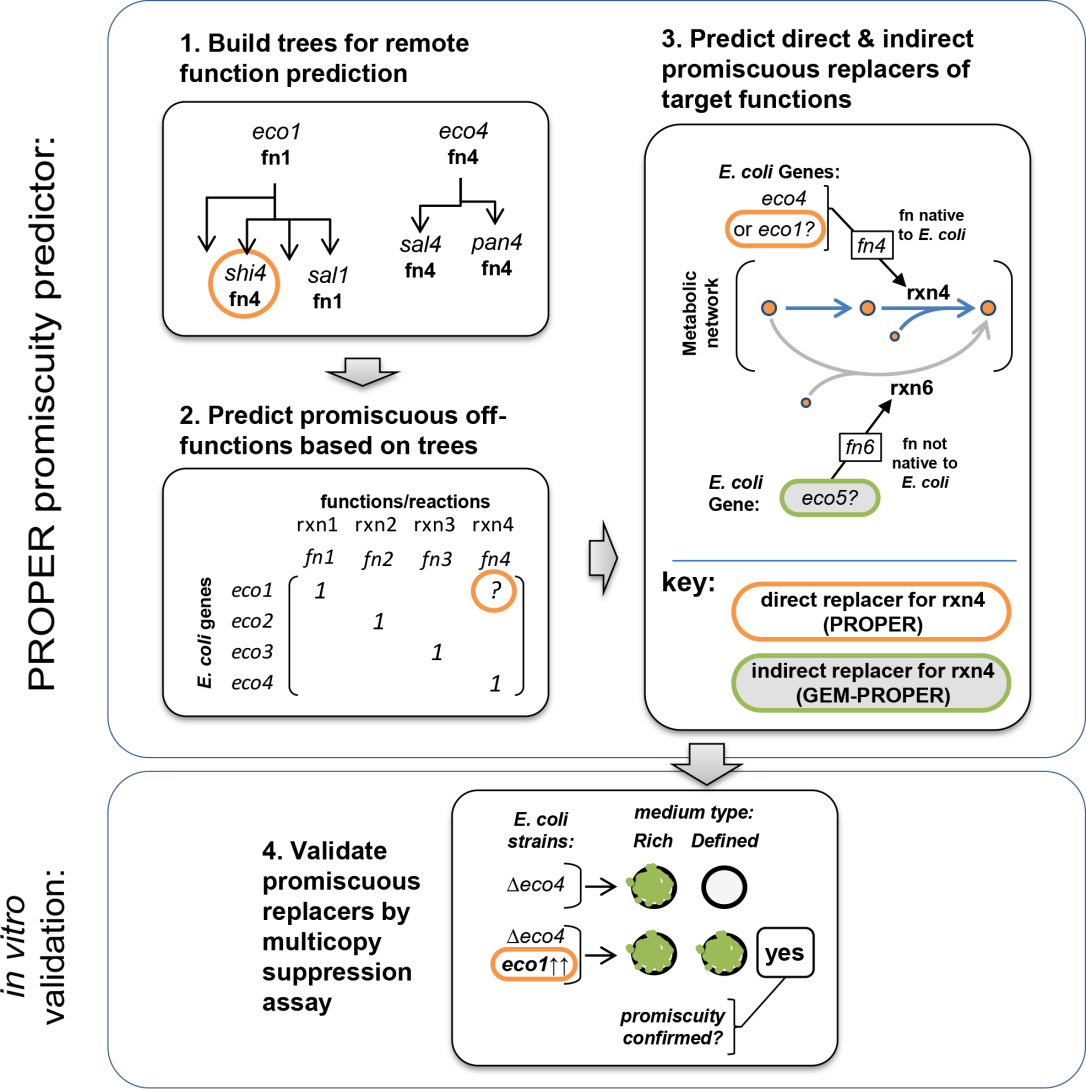
Schematic of prediction framework for promiscuous replacers. (1) Gene similarity trees are built around each gene in *E*. *coli*, including any distantly related gene in the RAST database. (2) A matrix is formed which links genes with their primary functions and also potential promiscuous functions. A gene (in this example, *eco1*) will take a potential secondary ‘promiscuous’ function in the matrix if its similarity tree includes any genes annotated with different functions (e.g., in this example, *shi4*, which encodes function fn4). (3) Cases in which a gene’s predicted promiscuous function is identical to the function of another gene in *E*. *coli* constitute predicted ‘direct’ target-replacer gene pairs (via PROPER). We also predict ‘indirect’ target-replacer pairs where a replacer bypasses the target’s function (via GEM-PROPER). (4) Promiscuous activity of a ‘replacer’ gene can be confirmed for target-replacer pairs in which the target is conditionally essential on a minimal medium, via the multicopy suppression assay.

We aimed to predict promiscuous functions of genes in a systematic and unsupervised manner, and specifically to do it on a genome-wide scale. We started by doing permissive PSI-BLAST based searches for distant gene similarities across RAST, a large consistent database of metabolic reactions and compounds that includes thousands of microbes [20]. This search allowed us to assign putative secondary functions for each initial “root” gene in the search (see Methods and Fig 1). We did this across metabolic genes in *E*. *coli*, which resulted in a set of phylogenetic trees, one for each root gene, that include up to thousands of genes from other bacteria along with their associated functions.

Using these trees, we searched for instances in which a gene in one of the trees had an assigned function different from the one that the RAST database assigns to the root gene. We restricted our search to genes whose primary functions are metabolic, as this is our focus of interest. Since our tree building method was more permissive than typical BLAST or PSI-BLAST, we considered these predicted functions as potential secondary functionalities that might only be effective at high expression levels or with slight mutations. We entered these cases in a large gene-by-function matrix.

To facilitate testing and validation, we used our promiscuity matrix to predict pairs of genes that correspond to those identified through an *in vitro* assay called Multicopy suppression [19]. Multicopy suppression tests ‘replacer’ genes for promiscuous activities that either directly (catalyze similar reactions) or indirectly (catalyze distinct reactions) replace the function of a ‘target’ gene and preserve growth. It proceeds in four steps: (1) a ‘target’ gene is identified in the bacterium of interest (target genes must be essential on a certain test medium [usually M9], but not on a rich medium); (2) a strain is created in which the target gene is inactivated (due to the condition in step 1, this strain is not viable on the test medium); (3) a library of native genes (hereafter called ‘replacers’) is individually over-expressed in the organism on high-copy plasmids; and (4) transformed strains are grown on the test medium, and survivors are noted as having identified successful target-replacer gene pairs. Importantly, the replacer genes are native to the genome of the organism, but their natural expression dosage is not sufficient to promiscuously support growth. Hence, replacer genes discovered through multicopy suppression are hypothesized to possess secondary, low-affinity/low-activity functions that compensate for the function of the target. We predict target-replacer pairs in two ways: direct replacements, and indirect replacements.

To predict direct replacements, we search our matrix for instances in which one ‘replacer’ gene in *E*. *coli* has a secondary function assigned through our method, which corresponds exactly to the function of some other ‘target’ gene that is also found in *E*. *coli*. We call this straightforward method the enzyme PROmiscuity PrEdictoR, or PROPER.

To predict indirect replacements, we use the added insight of a GEM of *E*. *coli* (obtained from SEED: [21]). The SEED *E*. *coli* GEM was used, as opposed to one of the highly curated *E*. *coli* GEMS such as [22], because of our requirement that metabolic reactions from the SEED database can be seamlessly added to the GEM to test their effect on cellular fluxes (which would first require a large reconciliation of nomenclature if using a non-SEED GEM). Specifically, we search for replacers that, through a promiscuous function identified in our matrix, metabolically bypass the need for the target. These predictions were done in three steps: (1) we knocked out the target gene in a GEM (this could only be done for genes that were conditionally lethal on M9 *in silico*); (2) we searched in our gene similarity trees for any *E*. *coli* genes that harbor non-*E*. *coli* promiscuous functions; (3) we added each of these promiscuous functions in turn to the GEM, and looked for any that rescued *in silico* growth. This GEM-based PROmiscuity PrEdictoR (GEM-PROPER) thus identified indirect replacer genes that act through metabolism (see panel 3 of Fig 1).

In all, PROPER predicted 2811 direct target-replacer pairs in *E*. *coli*, encompassing 794 metabolic target genes and 753 metabolic replacer genes (see S1 Table). GEM-PROPER predicted a total of 98 indirect target-replacer pairs in *E*. *coli* (see full list in S4 Table). Since direct replacers span a large portion of metabolism, we focus on these predictions for our large-scale analyses (i.e., the first several sections of the results). Afterwards, we shift our focus to GEM-PROPER, and present a detailed analysis of a novel indirect predictor that we validated.

### Systems-wide testing of PROPER

The number of direct replacers per target follows an exponentially decaying distribution (see S1 Fig), implying that promiscuous gene functions cluster around key target functions. The target genes with the most putative replacers are involved in fatty acid degradation or synthesis [*fadD* and *fadK* (degradation); *fabD* (synthesis)], or transport processes [*malF* (maltose/maltodextrin transport); *potC*, *ydcV*, *potB*, and *ydcU* (spermidine/putrescine transport); and *cysW* (sulfate transport)]. Fatty acid degradation and synthesis processes are known to involve enzymes with many potential specificities depending on only small alterations [23].

To validate our direct predictions, we collected results from two studies of multicopy suppression in *E*. *coli*, which between them had discovered 48 instances of multicopy suppression among 21 target genes [8, 19]. Both of these assays looked for multicopy suppressors of targets that were essential on M9 medium but not in a rich medium, and one used a combinatorial assay that assessed every gene in *E*. *coli* as a potential replacer [19]. All but 3 of the target genes for which replacers had been found were metabolic, supporting our focus on metabolism. In all, we predicted a total of 63 replacers for the 21 targets, versus the 48 found previously by multicopy suppression. The replacers we predicted overlapped with those found previously by 8 genes. Notably, for each of the 21 target genes, there were over a thousand metabolic genes that could serve as potential replacers; therefore, the chance of achieving this level of overlap between our predictions and those found experimentally through random guesses is extremely low (p = 4.5e-15 in a hypergeometric test).

As another control test, we performed a BLAST search of each target gene identified in Patrick *et*. *al*. against all metabolic genes in *E*. *coli*, and kept only those above the default threshold as potential replacers. This yielded 122 predicted replacers for the 21 target genes, among which none matched results from Patrick *et*. *al*., and only two overlapped with predictions made by PROPER (neither of those turned out true in our own experiments). This emphasizes the added value of PROPER over simple BLAST searches for finding find such low affinity promiscuous functions.

We also considered it possible that some of our target-replacer predictions are true, but cannot be verified using multicopy suppression because the target gene is not conditionally lethal (which is a necessary condition for doing a multicopy suppression experiment–see Fig 1, panel 4). As a quick test of this, we compared our direct predictions to the isozyme sets *aspC/tyrB*, *argD/astC/gabT/puuE*, and *gltA/prpC*, which were discovered in [17] as described in the introduction. Indeed, all 8 pairwise combinations of genes from within any of these 3 isozyme sets comes up as a *reciprocal* target-replacer pair in our direct predictions, meaning that either gene in each pair can play the role of the target or the replacer. That all of these pairs would come up in our predictions and, given that, that they would all be reciprocal are both highly unlikely by chance (p = 3e-19 and p = 3e-5 in hypergeometric tests, denoting that all pairs from [17] (a) overlap our predictions vs. a background of all-vs.-all pairings of our predicted targets and replacers, and (b) are reciprocal, assuming the same likelihood for any pair). This suggests that promiscuous functions that have enough activity to be considered isozymes (or, more specifically, to be defined so through the experimental pipeline in [17]) may tend to come up in our predictions as reciprocal, and suggests a complimentary association between our methods and those in the aforementioned work.

### Testing novel predicted direct replacers via new multicopy suppression assays

The random gene insertion method used by Patrick et al. to screen for potential target-replacer pairs [19] is demonstrably not comprehensive (e.g., 7 replacers were found for *pdxB* in [8]; none of these were reported in the Patrick study for *pdxB*, although *pdxB* was in the set of replacers that Patrick et al. tested). Hence, we next asked how many of our novel predicted target-replacer pairs (those that had not been previously found experimentally) may be true. To test this we developed a single-replacer multicopy suppression assay analogous to that used to validate individual results in the initial multicopy suppression study by Patrick [19]. This assay involves plating target-replacer strains (i.e., strains with the target knocked out, and the replacer over-expressed on a plasmid) on M9 medium, and observing how long it takes for colonies to appear. We judged successful target-replacer pairs as those that grew more robustly than an empty plasmid control (see Methods, as well as Supplementary methods in S1 Text for a fuller explanation of this assay).

Using these criteria, we verified 7 of the 8 target-replacer pairs that were identified in Patrick *et*. *al*. and predicted by PROPER (see S2 Table for full list of experimental results). We then assessed each of our 55 novel predicted target-replacer pairs for multicopy suppression, and found three of them to have replacer activity (*hisA*, *cysM*, and *metB* were viable replacers for *hisH*, *ilvA*, and *metC* respectively; see S2 Table). Thus, in all we validated 10 out of 63 direct replacement predictions, which gives a success rate of 20% (after eliminating 14 predictions for *ptsI* and *glyA* target genes, which were in practice untestable because the target knockout did not decrease growth from *wt* in our experiments; see S1 Text). Among the three validated novel target-replacer pairs we validated, the strength of the observed phenotype scaled with the strength of sequence similarity between the replacer gene and its match in the promiscuous gene tree (i.e., Panels 1–2 of Fig 1). Namely, *metB* fully restored growth in *ΔmetC* cells, while the less homologous *cysM* and *hisA* (in that order) less efficiently supported growth following target knockout (see S1 Text; also compare alignments in S4, S5 and S6 Figs). Thus, these examples suggest that higher similarity to the function-assigning gene corresponds to higher activity or affinity for that enzyme’s substrates, although a more extensive study of multicopy suppression phenotypes would be needed to uphold this observation generally.

### Indirect replacer predictions and follow-up mutagenesis experiments reveal *thiG* as having pyridoxal 5’-phosphate synthase activity

Among the 98 indirect target-replacer pairs we predicted, two were for the target *pdxB*, which is by far the most ubiquitiously replaced target found *in vitro* across published multicopy suppression datasets (in fact, *pdxB* is the sole focus of the Kim study: [8]). We confirmed our *in silico* predictions of these two target-replacer pairs using the manually curated *E*. *coli* model iAF1260 [22]. The target, *pdxB*, is a conditionally essential gene (essential on M9 medium, but not on rich medium) involved in the biosynthesis of pyridoxal 5’-phosphate (**P5P**), the active form of Vitamin B_6_. Vitamin biosynthesis reactions are good candidates for multicopy suppression, since vitamins are required in low amounts, so even a moderate flux through an over-expressed promiscuous enzyme could provide enough of the nutrient to enable growth [24]. P5P is an essential cofactor in all known living systems [25].

We tested our two predicted *pdxB* target-replacer pairs, and found one of them, *thiG* replacing *pdxB*, to be a true replacer [*∆pdxB/thiG* colonies were 1mm diameter, vs. 0.1–0.2mm diameter in *∆pdxB/empty*, after 3 days incubation in replicate experiments on M9 medium; see S2 Table]. The observed phenotype was consistent with previously reported *pdxB* replacers (1mm colonies of a *∆pdxB/replacer* strain after 1–2 days (3 cases) or 3–5 days (4 cases) at 37°C in equivalent growth conditions & temperature [8]). We explored the *thiG-pdxB* target-replacer pair in detail, as follows:

#### (1) Pathway and sequence similarity analysis to identify promiscuous function of *thiG*

**Fig 2:**
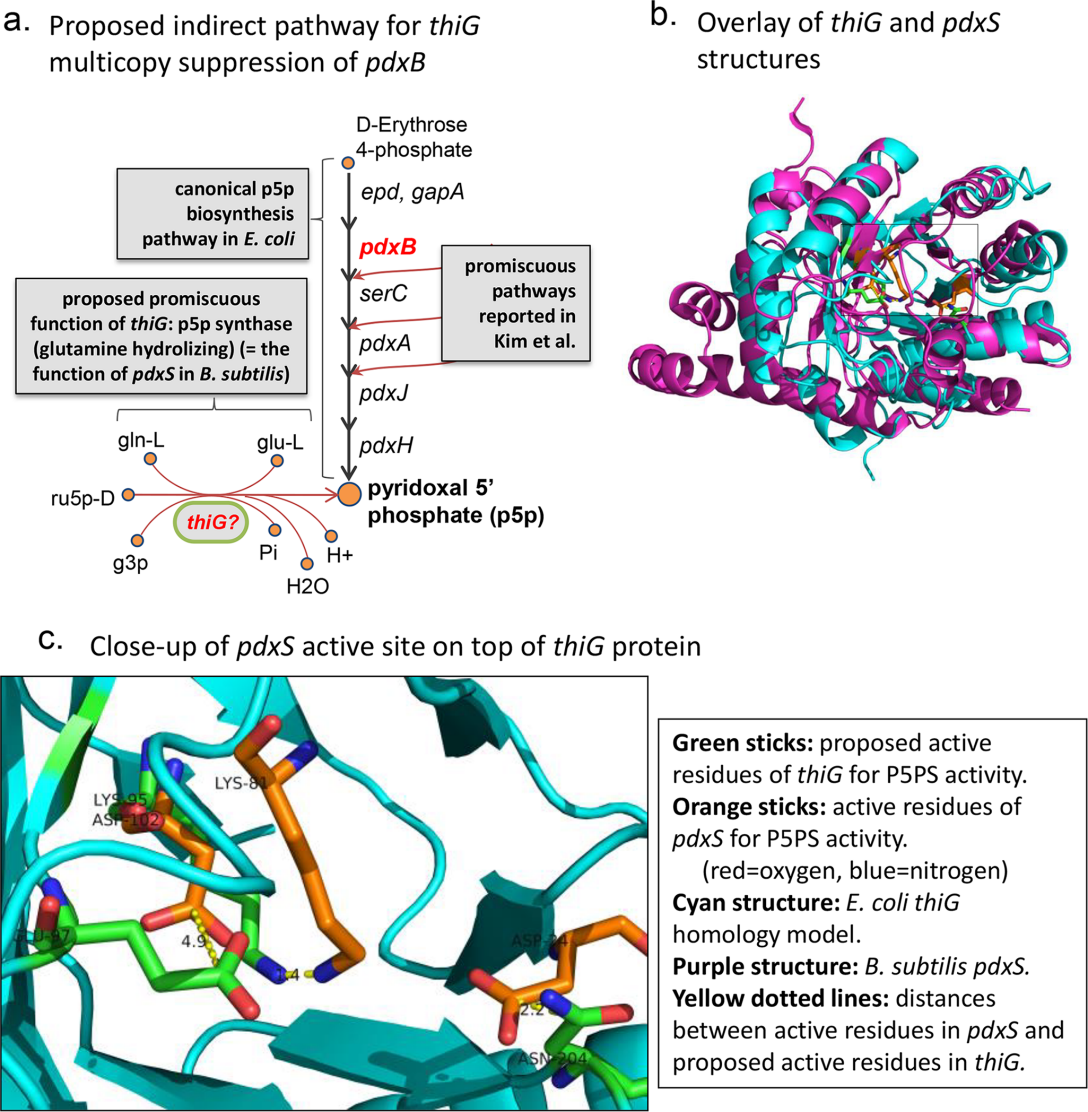
Proposed novel pathway for promiscuous production of pyridoxal 5’-phosphate. GEM-PROPER was used to predict the indirect target-replacer pair, *∆pdxB/thiG*, which we then confirmed with experiments. The predicted secondary function of *thiG* is pyridoxal 5’-phosphate synthase (P5PS), which would bypass the known 6-enzymatic-step pathway for production of p5p in *E*. *coli*. (a) The two alternative pathways, along with known promiscuous pathways in *E*. *coli* for producing p5p after *pdxB* knockout (as reported in Kim: [8]). Abbreviations are: ru5p-D = D-Ribulose 5-phosphate; gln-L = L-glutamine; g3p = Glyceraldehyde 3-phosphate; glu-L = L-glutamate; Pi = Phosphate. (b) Structural alignment of a homology model of *thiG* (for *E*. *coli*, based on crystal structure of *thiG* from *B*. *subtilis*) with a crystal structure of *B*. *subtilis pdxS*, the gene that (in complex with another gene, *pdxT*) performs the P5PS function in *B*. *subtilis*. The proteins share the TIM barrel fold. (c) Close-up of the structural alignment in (b), focused on the active site of *pdxS* and the residues of *thiG* that we propose perform the *pdxS* function. The location of the close-up is shown with a box in (b).

Among the 7 multicopy suppressors (i.e., replacers) previously reported in Kim et. al. for *pdxB* [8], all were inferred to rescue growth of *ΔpdxB* by contributing downstream intermediates to the pyridoxal 5’-phosphate synthesis pathway, thus bypassing *pdxB* (see Fig 2A). In contrast, the predicted multicopy suppression activity of *thiG* that we confirmed experimentally suggests that ThiG promiscuously assumes the PdxS function ‘Pyridoxal 5’-phosphate synthase (glutamine hydrolyzing)’ (hereafter referred to as: **P5PS**), a reaction that has been described in *Bacillus subtilis* and other organisms and that completely bypasses the 6-step Pyridoxal 5’-phosphate synthesis pathway known in *E*. *coli* (see Fig 2A) [25, 26].

Our prediction of *thiG* supporting Vitamin B_6_ biosynthesis is based on similarities between a portion of *thiG* and a portion of *pdxS* from *Staphylococcus haemolyticus*, as found in our gene similarity trees. *pdxS* in *Staphylococcus haemolyticus* was annotated as such based on high similarity (81% identity over 98% of the genes) with the well-studied *pdxS* gene in *B*. *subtilis* (e.g., see [25] and [27]). A BLASTP gene alignment of *B*. *subtilis pdxS* versus *E*. *coli thiG* confirms that the genes share a region of moderate similarity, which contains conserved residues of *pdxS* [25] (see S7A Fig).

PdxS has been shown to dimerize with PdxT to form a complex that catalyzes the P5PS reaction, with PdxS and PdxT acting as ‘synthase’ and ‘glutamine amido transferase’, respectively. In *E*. *coli*, glutamine amidotransferases exist that have some sequence similarity to *pdxT* (e.g., the gene *guaA*, GMP synthetase, which BLASTP reveals to have 29% sequence identity with *pdxT* over 77% of the *pdxT* gene). Notably, our list of indirect replacers for *pdxB* also included *hisH*, with the predicted function of *pdxT*. Thus, *hisH* is a strong candidate for functionally complimenting the potential *pdxS* function of *thiG*. However, *pdxS* is also present in the genomes of certain organisms in the absence of *pdxT*, and it has been hypothesized that it might operate in these organism as an ammonium-dependent synthase of P5P, thus bypassing the need for the glutamine amidotransferase subunit *pdxT* [26]. It is therefore plausible that the low affinity ‘replacer’ function of *thiG* produces P5P either with or without an amidotransferase (using glutamine, ammonium, or some other substrate), and that this is the mechanism by which increased ThiG restores growth in *ΔpdxB* cells.

#### (2) 3-D structural alignment to determine putative *thiG* promiscuous active site for P5PS activity

We performed a 3-D alignment of ThiG and PdxS protein structures (*E*. *coli* ThiG homology-model structure was built using the crystal structure from *B*. *subtilis* as template; PdxS structure was taken from *B*. *subtilis*; see methods). This structural superimposition revealed that *E*. *coli* ThiG and *B*. *subtilis* PdxS share the same fold [TIM barrel: (βα)_8_], and revealed a potential overlap of ThiG residues with the PdxS active site: Lys-95, Asn-204, and Glu-97 from the *E*. *coli* ThiG homology-model lie within 1.4, 2.2, and 4.9 angstroms respectively from the catalytic residues Lys-81, Asp-24, and Asp-102 in PdxS (distances were determined between alpha-carbons of the carboxylic/amide groups in the active sites of the Asn, Glu, and Asp, or between Nitrogens in the active group in the case of Lys—see Fig 2B and 2C). These distances are well within the error of homology models [28], and we thus hypothesize the above-mentioned amino acids can perform the PdxS function. Multiple views of this alignment are shown in Figs 2B and 2C and S8. One of the residues in ThiG that we propose to be active in its promiscuous PdxS-like function (Glu-97) is one of the two natural ThiG dyad active site residues used during thiamine biosynthesis (see S7B Fig), but the other two proposed residues are not [29]. Thus, the proposed PdxS activity of ThiG is not solely a repurposing of the original active residues. We found that all three proposed residues are more evolutionarily conserved than neighbor residues, suggesting a functional role (ConSurf calculations with default parameters and max homology threshold of 70%; see S9 Fig) [30].

#### (3) Inactivation of putative *thiG* promiscuous active site to test if that removes *pdxB* replacement activity

To validate the putative secondary triad active site of ThiG (i.e., the residues Lys-95, Asn-204, and Glu-97), we tested whether inactivation of the predicted active site would eliminate the ability of ThiG to replace PdxB *in vitro*. We therefore constructed an overexpression plasmid containing a mutated copy of *thiG* in which we performed alanine substitution of Lys-95 and Glu-97 (L95A, E97A). The third residue of the proposed active site, Asn-204, was not altered since, as noted, this residue comprises part of the primary ThiG dyad active site. We thus created a mutated form of *thiG* (*thiGmut*) with the proposed secondary active site disturbed, but the site for the primary activity of *thiG* unaffected.

To investigate the effect of *thiGmut* overexpression on the growth of Δ*pdxB* cells in minimal media, we transformed Δ*pdxB* cells with plasmids encoding IPTG-inducible copies of *thiGmut* (*ΔpdxB/thiGmut*), *thiG* (*ΔpdxB/thiG*), *pdxB* (*ΔpdxB/pdxB* rescue plasmid positive control), as well as the empty vector (*ΔpdxB/empty* negative control). To determine the optimal M9 glucose media conditions for growth comparison, we performed a checkerboard dilution assay in deep-well microplates in which we individually assessed the growth of each strain across a matrix of varied IPTG and ammonium nitrate (NH_4_Cl) concentrations to identify synergistic combinations of inducer and nitrogen source, respectively. The nitrogen source (NH_4_Cl) was varied because we observed that vitamin B6 is used heavily as a cofactor in enzymes that participate in amino acid metabolism (S10 Fig), so we hypothesized that altered levels of nitrogen consumption might have some effect on the demand for p5p, and thus might influence the signal to noise ratio in validation experiments for ThiG secondary activity.

**Fig 3:**
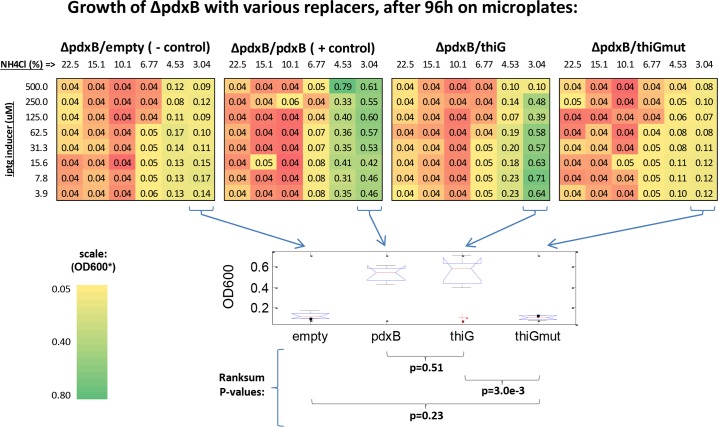
Inactivating the *thiG* proposed secondary active site removes its ability to replace *pdxB*. Four strains (*ΔpdxB/empty* [- control], *ΔpdxB/pdxB* [+ control], *ΔpdxB/thiG*, *ΔpdxB/thiGmut*) were grown for 96 hours in deep-well microplates, in which a checkerboard matrix of varied IPTG (inducer) and NH_4_Cl (nitrogen source) concentration in M9 glucose were assessed. Values shown are representative OD_600_ readings at 96 hours post-inoculation. Additional OD_600_ data are provided in S5 Table. Box plots of OD_600_ values at ~3% NH_4_Cl (across IPTG concentrations) are shown in lower panel, along with the results of Ranksum tests of OD_600_ values between relevant strain pairs.

Strikingly, we found that IPTG and NH_4_Cl combinations that supported the most robust growth of our *ΔpdxB/pdxB* control were also supportive of *ΔpdxB/thiG* at 48 and 96 hours post-inoculation (Figs 3A and S11, S5 Table). Conversely, we observed that *ΔpdxB/thiGmut* and *ΔpdxB/empty* similarly exhibited poor growth across all media conditions. Ranksum tests of optical densities (OD_600_) for all strains grown at the experimentally observed optimum NH_4_Cl concentration (~3%), across all IPTG concentrations, revealed highly significant differences between growth of the *ΔpdxB/pdxB* and *ΔpdxB/thiG* group versus the *ΔpdxB/thiGmut* and *ΔpdxB/empty* group after 96 hours (p< = 4.7e-3), but no significant differences within these groups. Importantly, these findings were consistent with subsequent batch culture growth experiments performed using the optimized concentrations of 10 μM IPTG and ~3% NH_4_Cl in M9 glucose (S11 Fig). These data strongly indicate that inactivation of our proposed secondary active site in *thiG* negatively impacts the ability of *thiGmut* to promiscuously replace *pdxB in vitro*.

## Discussion

Identifying promiscuous gene functions is a fundamental task in biology. Promiscuous functions have been causally associated with the evolution of new gene functions [24, 31], and may have contributed to the evolution of resistance to antimicrobials and other stresses [7]. Here, we present a new method termed PROPER that uses iterative PSI-BLAST-based phylogenetic trees for predicting potential promiscuous functions based on weaker similarities than are typically considered in assigning gene functions. Based on these predictions, we experimentally validate 4 novel target-replacer pairs, one of which (*thiG* replacing *pdxB*), was found by coupling our promiscuity predictor with a GEM. Finally, we predict and then experimentally validate the promiscuous active site of *thiG* for this replacing activity, revealing a striking new promiscuous route for the production of an essential nutrient, p5p, in *E*. *coli*.

While sequence similarity-based methods (e.g., BLAST) are used ubiquitously to determine primary gene functions, their ability to call promiscuous functions is unclear. Our method gives a first estimate, as we were able to experimentally validate around 20% of direct target-replacer pairs we could test in the lab (This is 100x higher than expected if guessing target-replacer pairs randomly). It is likely that the number of target-replacer pairs we validated is an underestimate of true promiscuity, as some true replacers might not show during multicopy suppression due to incorrect expression levels (e.g., due to non-optimal IPTG concentrations), insufficient effects of target knockout, or other confounding factors. Notably, many of our predictions might be correct even though they cannot be directly validated through multicopy suppression (due to the necessity in multicopy suppression that the target gene is conditionally essential). Our comparison to the work of [17] emphasizes this point, as several of our predicted target-replacer pairings came up in that work, regardless of the fact that the target genes examined in that study are not conditionally essential. The promiscuous functions we predict thus might represent a large underground repertoire that could be activated under the right kinds of selective pressure.

Our method for predicting promiscuous gene functions utilizes the RAST database and automatically constructed metabolic models from SEED, resources that include thousands of bacterial and archaeal species. While manually curated GEMS for *E*. *coli* are available (e.g., [32]), we chose to use the SEED GEM because it interfaces smoothly with the SEED and RAST databases, which was necessary for implementing GEM-PROPER without needing to reconcile thousands of metabolite and reaction names with an outside model. A second benefit of this choice is that although we focus here on predicting promiscuous gene functions in *E*. *coli*, our methods are generic and may easily be extended to any other organism in RAST. This could, for example, aid in determining resistance or adaptation mechanisms across major pathogenic strains, which could then be targeted in new cures.

In all, this work constitutes the first ever genome-wide prediction of metabolic gene multicopy suppression, and the first integration of such predictions with a GEM to achieve network-reliant indirect predictions. We begin with an unsupervised, large-scale method to predict enzyme promiscuity, and finally dial in on and then experimentally verifying a key prediction of biological significance in *E*. *coli*. Without such a systems approach, it would have been extremely difficult to identify *thiG* as a candidate replacer for *pdxB*. Thus, this study serves as a successful example where systems biology can provide a roadmap for biological investigation, identifying the most promising targets to then follow up on with more costly and time-consuming experiments.

## Materials and Methods

### Implementation of PROPER

PROPER proceeds in three steps, as outlined in Fig 1: (1) building gene similarity trees for all genes in *E*. *coli*; (2) using these trees to build a matrix of promiscuous gene functions; and (3) applying this matrix to determine potential promiscuous gene replacers for target genes of interest. In GEM-PROPER, step (3) is replaced by a GEM-based approach, in which we search for promiscuous functions (from step 2) that can rescue *in silico* growth of a GEM model after knockout of the target gene. Here, we describe these steps in detail:

#### Step 1: Building the enzyme promiscuity trees

To identify as many promiscuous gene functions as possible, we performed an unsupervised iterative PSI-BLAST based search for distant sequence relationships between genes in E. coli and all other genes in the SEED database. Our method involved five steps: (1) align the current query sequences; (2) cluster the aligned sequences to reduce sampling bias in sequencing; (3) trim aligned representatives to eliminate non-conserved ends; (4) use trimmed alignments in a PSI-BLAST search against the non-redundant protein database in SEED; and finally, (5) keep PSI-BLAST hits that match certain criteria, as outlined below. These hits would be kept for the next round of PSI-BLAST, and the process would continue for three rounds with increasingly more relaxed thresholds for keeping PSI-BLAST hits.

The intuition behind this approach was to use iterative trimming to build a compact protein profile that is effective at recruiting distant homologs. These profiles differ from individual protein domains such as some CDDs in that they capture the conserved arrangement of multiple domains in related protein sequences. The parameters used in this protocol were manually tuned to facilitate inspection of related protein functions in the same tree for expert annotators at SEED. The detailed protocol and parameters are provided in the supplementary materials (S1 Text). For the last PSI-BLAST search, a combination of the following cutoff criteria was used:

1. uncovered region of the query sequence < = 100
2. the query sequence is at least 60% covered
3. sequence percent identity > = 0.15
4. sequence percent positive match > = 0.30 [This is the percentage of aligned bases with positive scores from the substitution scoring matrix indicating conserved changes (identical pairs included)].

As PSI-blast itself can do iterative search, the main difference in our approach was trimming and clustering. By trimming we eliminated non-conserved ends, so the profile is more compact and more effective at recruiting distant homologs during each search. The clustering step keeps only the representative sequences in the profile, thus alleviating some of the sampling bias, i.e., so that the profile is not dominated by *E*. *coli* variants of the protein. We use MAFFT [33] for alignment and FastTree [34] for tree building.

This whole procedure provided a tree for each gene in *E*. *coli* that links to other genes with any sequence similarity, which were then candidates for assigning potential (predicted) promiscuous functions to the root gene.

#### Step 2: Building a matrix of promiscuous gene functions

Genes in RAST are annotated with functions, which then (in the case of metabolic genes) link to metabolic reactions. After a few attempts to set distance thresholds in the trees for assigning functions, we found that including all function in the tree as a potential promiscuous function gave optimal results. Therefore, all functions existing in the tree of a root E. coli gene are considered potential promiscuous functions, as are their metabolic reactions. In cases where a gene is promiscuously associated with only one subunit of an enzyme complex, we predicted that entire function is associated with the root gene. We entered these gene-function promiscuous pairings into a matrix for further analysis.

#### Step 3 in PROPER: Determining direct target-replacer pairs

To search for direct target-replacer pairs, we identified cases in our matrix in which any of a ‘replacer’ gene’s promiscuously assigned functions is the same as the primary annotated function of a ‘target’ gene of interest.

#### Step 3 in GEM-PROPER: Determining indirect target-replacer pairs

To search for indirect target-replacer pairs, we identified cases in our matrix in which any of a ‘replacer’ gene’s promiscuously assigned functions can metabolically bypass the primary annotated function of a ‘target’ gene of interest. This analysis could only be done for target genes that are conditionally essential in silico, i.e., whose knockout causes in silico death on M9 medium but not on rich medium.

The GEM used was a genome-scale metabolic model for *Escherichia coli K12* taxon ID 83333.1 as downloaded from SEED [21]. Target genes for which Patrick had found replacers were assessed for M9 medium conditional essentiality *in silico*, and the subset of Patrick targets that were also conditionally essential *in silico* were kept for further analysis. All essentiality tests were done using Flux Balance Analysis testing for a nominal production of biomass [35]. Metabolic reactions associated with functions in our enzyme promiscuity matrix were added to the GEM individually after knockout of each of the targets to assess if *in silico* growth was rescued. Cases in which the indirect replacer gene (i.e., the one associated with the rescuing reaction) rescued *in silico* growth were considered predictions of indirect replacement.

### Experimental validation of promiscuity predictions via multicopy suppression

Multicopy suppression is an established assay that has been described elsewhere (see panel 4 in Fig 1, and, e.g., [8, 19]). In our experiments, we tested specific target-replacer pairs in the manner used by Kim to validate target-replacer pairs they identified in their large-scale screen. Namely, we inserted a plasmid containing the replacer gene with an IPTG-induced promoter into a KEIO strain with the target gene knocked out, and assessed whether this target-replacer strain grew better than the target knockout strain without the replacer. Specifics of strain construction and the experiments follow:

### Bacterial target, target-replacer, and control strain construction

*E*. *coli* strains containing specific gene knockouts were obtained from the KEIO collection [36], which contains single-gene knockout strains for the majority of genes in *E*. *coli*. Plasmids for IPTG-inducible expression of predicted replacer genes were obtained from the ASKA collection [37], which contains plasmids that overexpress nearly every individual gene in *E*. *coli*. Both collections are obtainable from the National Bioresource Project at the National Institute of Genetics, Japan. KO target strains were made electro-competent (4x washes with ddw/10% glycerol) and transformed with ASKA plasmids over-expressing the corresponding predicted replacer gene.

When we were developing our assay, we found that knockout colonies usually grew after some amount of time on M9 medium regardless of whether the replacer gene had been added to the plasmid, despite the fact that each of the target strains we tested was previously reported to be non-viable on M9 medium. The previous study by Patrick [19] had not plated background target-deleted strains that had empty plasmids inserted. Due to the eventual growth in most strains, this was an important negative control for judging that a replacer was actually compensating for the loss of the target and improving growth (and was done in Kim: [8]). Therefore, for each KO strain we additionally transformed with an empty ASKA plasmid as a negative control. We judged a strain to be a true replacer only if it came up consistently stronger and/or earlier than the strain carrying the empty plasmid.

### Verification of target identity (knockout location) and plasmid insertion

In the KEIO collection each knocked out gene is replaced by the kanamycin resistance gene (*KanR*). Thus we verified correct location of the knockout in each target strain by amplifying and sequencing the area directly flanking the kanamycin insertion, using a kanamycin universal primer and a strain-specific reverse primer located downstream of the knocked out gene. Since the orientation of the kanamycin insertion depends on the orientation of the original knocked out gene, the *KanR* primer 5’-ATATTGCTGAAGAGCTTGG was used when the original gene had been forward coded and the *KanR* primer 5’-AATGAACTCCAGGACGAG was used if the original gene had been reverse coded. Correct sequence of the replacer gene was verified by amplifying and sequencing the ASKA plasmid insert (forward: 5′-ATC ACC ATC ACC ATA CGG AT; reverse: 5′-CTG AGG TCA TTA CTG GAT CTA).

### Target-replacer plating experiments

Starter cultures of each target-replacer (TR) pair were grown overnight in LB+CAP, washed and serially diluted in saline (0.9% NaCl). Aliquots (100 ul) of 10^−6^ dilution were plated on LB+chloramphenicol and on M9-glucose-kanamycin-chloramphenicol (1× M9 salts, 2 mM MgSO4, 0.1mMCaCl, 0.4% glucose, 34 μg/mL chloramphenicol, 30μg/ml kanamycin) containing IPTG. All TR pairs were plated on both M9 with 50μM and 125μM IPTG; TR pairs of particular interest (*hisA/∆hisH*, *cysM/∆ilvA* and *thiG/∆pdxB)* were plated on 250μM as well. In each TR plating experiment, the empty and *frvX* negative controls were plated alongside the TR pairs. Plates were wrapped in nylon to avoid dehydration, incubated at 37 and monitored during the next three weeks. Nearly all negative control plates showed some growth phenotype, ranging from very strong (normal sized colonies within 3 days) to very weak (pinpoint colonies after 3 weeks; S3 Table). Thus, a TR pair was considered valid only if colonies consistently formed at a higher rate than in both negative control plates. Each plating experiment was repeated 2–3 times.

### Growth in liquid M9

Growth of TR pairs that showed exceptionally high growth on the negative control plates (*ΔglyA*, *ΔptsI* and *ΔpabB*) was also monitored in liquid culture. Cultures in LB+CAP were washed once in saline and re-suspended at a dilution of 1:100 into M9-glucose-kanamycin-chloramphenicol minimal media supplemented with IPTG (50 μM). Growth measurements were performed in a 96-well plate incubated for 19–24 h at 37°C in a temperature-controlled plate reader with continuous shaking (ELX808IU-PC; Biotek), and OD595 was monitored every 15 min. Each TR pair was loaded into 2 duplicate wells. Growth of the negative controls (empty and *frvX* plasmids) for each target strain was likewise monitored during every run.

### Verifying non-heterogeneity of ∆pdxB

It was reported in [8] that knockout strains of *pdxB* sometimes display a heterogeneous phenotype, with some growing on minimal medium and others not. To be sure that this was not a factor in our experiments verifying the *thiG/∆pdxB* replacer-target pair, we grew individual colonies of *∆pdxB* and confirmed that there was no heterogeneity in their growth on M9 medium.

### Microwell plate experiment and batch culture for thiG replacement of pdxB

Overnight cultures of each strain were grown in LB with 30 μg/mL kanamycin (*ΔpdxB* cells) and 25 μg/mL chloramphenicol (ASKA plasmids) selection. Cells were washed in 1x PBS and diluted 1:100 into M9-glucose-kanamycin-chloramphenicol for plate seeding; M9 was prepared without a nitrogen source. A checkerboard matrix was generated in 2mL deep-well, 96-well assay plates by serial dilution of NH_4_Cl (22.5% w/v maximum concentration) across plate columns and IPTG (500 μM maximum concentration) across plate rows. Wells were uniformly inoculated with cells, and each well contained a final volume of 1.2 mL. Plates were sealed with gas-permeable membranes and grown in a light-protected, microplate incubator shaker at 37°C and 700 RPM; 700 RPM was determined to be equivalent to 300 RPM in a standard incubator shaker. Samples for OD_600_ measurements were taken at designated timepoints using a SpectraMax M5 microplate multimode plate reader (Molecular Devices), and the gas permeable membrane resealed after each timepoint. 100 μL samples were taken for OD_600_ measurements to minimize the total volume loss.

For batch culture experiments, cells were prepared from overnight cultures as described above. Cells were diluted 1:100 in 30 mL of M9-glucose-kanamycin-chloramphenicol, containing ~3% NH_4_Cl (w/v) and 10 μM IPTG, in 250 Erlenmeyer flasks. Cultures were grown at 37°C and 300 RPM in an incubator shaker, with samples for OD_600_ measurements taken at designated timepoints.

### Structural alignment of *pdxS* and *thiG*

The X-ray structure of pdxS (from *B*. *bacillus*) in complex with pdxT, was downloaded from the pdb database, and the residues of the pdxS active site were identified from the publication (active residues are: K149, D102, K81 and D24) [38]. Multiple *pdxS* units from the multimeric structure were overlaid and were found to be coincident (as can be seen in Figs 2B and S8). No structure of *thiG* was available from *E*. *coli*, so a homology-model was built using the SWISSMODEL pipeline (http://swissmodel.expasy.org/; [39]) based on the *thiG* template from *Thermus thermophilus* (51.98% seq identity; PDB ID 2htm [40]). The structures of *pdxS* were subsequently aligned with that of *thiG* using pyMol [41].

### Effects of IPTG on replacers in multicopy suppression assays

Low (50μM) IPTG concentration was sufficient to induce all of the replacers. Interestingly, increasing the IPTG concentration affected growth sometimes positively and sometimes negatively, depending on the specific strain. Increasing IPTG concentration up to 250μM caused increases in colony size and number, and decreases in incubation times, for *hisA/∆hisH* and *cysM/∆ilvA*. Conversely, growth of *purE/∆purK*, one of the target-replacer pairs predicted by both Patrick and us, was almost entirely inhibited when IPTG concentration was increased to 125uM. These results illustrate that over-expression can also have deleterious effects [42]. The optimal level of expression (and hence the optimal IPTG concentration) depended on the specific target-replacer combination. In agreement with this, in several target strains, over-expression of the randomly chosen gene *frvX* caused a decrease of the background seen in the empty plasmid control (see S3 Table).

## Supporting Information

S1 Text Supplementary methods and results. (DOCX)

S1 Fig Number of replacers per target. (TIF)

S2 Fig Promiscuous functions of metabolic genes tend to be metabolic. All target-replacer pairs predicted by our method were assessed for how often a metabolic target paired with a metabolic replacer, with a non-metabolic replacer, a non-metabolic target with a metabolic replacer, etc. We found that (A) Replacer genes with metabolic primary functions take more metabolic targets than do replacer genes with non-metabolic primary functions; (B) Replacer genes with non-metabolic primary functions take more non-metabolic targets than do replacer genes with metabolic primary functions; and (C) The number of targets replaced by replacer genes with non-metabolic vs. metabolic primary functions is the same.
(TIF)

S3 Fig Statistics of gene similarity trees. Histograms are shown of: (A) number of genes represented in each tree; (B) number of unique organisms represented in each tree; (C) number of distinct functions represented in each tree; and (D) number of E. coli metabolic functions present in each tree.
(TIF)

S4 Fig BLASTP Alignment of hisA and hisH. (TIF)

S5 Fig BLASTP Alignment of cysM and ilvA. (TIF)

S6 Fig BLASTP Alignment of metB and metC. (TIF)

S7 Fig Sequence analysis of thiG. (A) E. coli thiG is aligned with pdxS, the gene whose function it putatively performs promiscuously. Key residues in both genes are marked, as per the key at the bottom. Two alignments are shown as they came up in BLASTP sequence alignment. (B) The active residues of thiG for its primary function are shown, along with putative active residues for the pdxS function.
(TIF)

S8 Fig Structural alignments of thiG and pdxS. Multiple alignments are shown.
(TIF)

S9 Fig Evolutionary conservation of the putative pdxS residues in thiG. The residues in thiG that we predict can perform the pdxS function show a high degree of conservation, as shown in the plot. This was generated from the output of ConSurf, set with default settings except for a maximum cutoff of 70% homology in the sequences to be aligned (higher cutoffs led to less resolution in distinguishing how conserved the key residues are versus their neighbors).
(TIF)

S10 Fig Frequency of usage of Vitamin B6 as a cofactor across pathways. (DOCX)

S11 Fig Inactivating the thiG proposed secondary active site removes its ability to replace pdxB, 48 hour timepoint. Results analogous to those shown in Fig 5 of the main text are shown, but at the 48 hour timepoint (instead of 96 hours as shown in the main text). (A) Ranksum test across all IPTG concentrations in the microwell plate experiment (equivalent of that shown in Fig 6, but for the 48 hour timepoint instead of 96 hours). (B) Results of a separate batch culture experiment, with 5–8 replicates per group, which show the same trend of the microwell plates from subfigure A. P-values are from t-tests across replicates of each strain grown in identical condition (4% NH4Cl and 10uM IPTG with otherwise minimal M9 media). #OD600 values in (A) were measured using 100uL of culture, where 300uL of culture would be the equivalent of a normal 1mL cuvette.
(DOCX)

S1 Table Direct replacer stats. (XLSX)

S2 Table Experimental results. (XLSX)

S3 Table Target backgrounds. (XLSX)

S4 Table Indirect replacers. (XLSX)

S5 Table Microwell experiment. (XLSX)
