# Supplementary material for: Systems-Wide Prediction of Enzyme Promiscuity Reveals a New Underground Alternative Route for Pyridoxal 5’-Phosphate Production in E. coli
Source: PLoS Comput Biol. 2016 Jan 28;12(1):e1004705. doi: 10.1371/journal.pcbi.1004705 (PMC4731195; doi:10.1371/journal.pcbi.1004705)
Supplement: S1 Text — (DOCX) [file pcbi.1004705.s001.docx]

# Supplementary Information:

## Supplementary methods

### Development of the assay to call target-replacer pairs

In designing our assay, we aimed to replicate protocols that have been employed elsewhere by other groups (notably in Patrick et al. ^1^, Soo et al. ^2^, and Kim et al. ^3^). The controls we used were also used previously in the Kim paper. Since several of the results from Patrick et al. that we retested in our work required a comparison between the replacer strain and the background (control) in order to call a replacement phenotype (3 out of 8 target-replacer pairs that Patrick had reported were of this type), and a considerable number of the target knockout strains from Patrick grew on minimal media if left long enough (despite being reported to not grow in Patrick and in the essentiality datasets from the Keio collection), we set our assay up such that we would consider a strain a successful target-replacer pairing if it grew better than the background strain, which we reported as a difference in colony size at a certain timepoint (in practice, the colony sizes were monitored each day until the colonies they were large enough that a call could be made; this was done in triplicate when appropriate, e.g., when we called a new target-replacer pairing). In the case of target knockout strains with extremely high growth, we also employed an alternative assay in which we compared the putative replacer strain to the empty plasmid background in liquid cultures, and compared the growth curves (this is explained in the methods). These results are not explored in the paper simply because none of those strains showed notable replacer activity (the target genes tested this way were: *glyA*, *ptsI*, and *pabB*).

In the case of the *ΔpdxB/thiG*, we did further assays beyond the single timepoint colony size assay (as shown in the last figure of the main text). These tests confirmed the original assay result, which had been based on colony size as done for the other strains. A comparison of the colony sizes we achieved after growing the *ΔpdxB/thiG* strain for three days versus colony sizes found for *ΔpdxB/replacer* strains in Kim et al. (as reported in Table II in the main text of that paper) puts our *ΔpdxB/thiG* phenotype in the midrange of the reported replacer phenotypes from Kim, so we deemed that using a stricter assay (e.g., saying that there must be no growth of the control strain whatsoever in order to call a valid replacer phenotype), or a more permissive assay (i.e., not checking for a difference between the replacer strain and the control strain, but accepting any replacer strain that grew as a valid replacement) would both lead to bad calls, and we chose the middle ground described previously (i.e., seeing a difference in growth between the replacer strain and the control). From our observations just described with comparing our results to the Kim and Patrick results, we deemed the comparison of colony sizes a reasonable way to make calls of valid target-replacer pairs.

## Supplementary results

### Metabolic versus non-metabolic targets/replacers

Although we focus generally on only metabolic replacers and targets (as stated in the main text), we wanted to be sure that we were predicting functions in the correct areas of cellular function. Therefore, we calculated direct replacers for both metabolic and non-metabolic genes for comparison. Our predictions indicate that replacer genes whose primary functions are metabolic replace significantly more metabolic targets than do replacers whose primary functions are non-metabolic (p=2.5e-95 in ranksum test; see Figure S2a). On the other hand, replacers whose primary functions are non-metabolic are more likely than those whose primary functions are metabolic to replace non-metabolic genes (p=2.0e-24 in ranksum test; see Figure S2b). There is no difference between metabolic and non-metabolic genes overall in the total number of *E. coli* targets they replace (p=0.79 in ranksum test; see Figure S2c). This indicates that most of the genes predicted to be replaced by metabolic genes are metabolic, and that this trend is not simply reflective of a difference in the total number targets of metabolic versus non-metabolic replacers.

### Several target-replacer pairs from Patrick are not active above background

In our initial validation experiments, we found that one of the replacers both predicted by us and reported by Patrick, *ltaE/∆glyA*, was not active above background (indeed, *empty/∆glyA* and several other target knockout strains showed no growth suppression on M9 in our experiments, suggesting that Patrick’s KEIO wildtype may have behaved differently than ours). Furthermore, we noticed that the replacers we predicted correctly from Patrick tended to be among those reported to come up the most quickly (p=0.03 in 1-sided ranksum test). Since the Patrick study did not include an empty plasmid background control, this begged the question of whether other replacers reported by Patrick might also have been false positive calls. We especially were interested in strains in which we saw the background come up very quickly, such as the aforementioned target strain *glyA*, or in which Patrick had reported that the replacers took a long time to come up (for example, the strain *chbA/ΔpurF*, which reportedly took 21 days). In all, we tested 5 extra strains that we had not predicted to be multicopy suppressors, but that had been reported as successful multicopy suppressors by Patrick, and that we hence suspected might have been false positive calls in Patrick et al.’s experiments ^1^. Of these, only one (*purF/ΔpdxB*) turned out to be a real replacer according to our experiments (see Supplementary Table S2). This emphasizes the importance of good controls in determining promiscuous functions, and increases the apparent accuracy of our predictions.

### *in vitro* growth of newly validated direct replacers scales with their homology in promiscuity trees

The measure of a successful multicopy suppression event in our *in vitro* assay for validating direct promiscuity predictions is ‘above background activity’, which meant different things for different strains. *∆hisH/hisA* came up only after 8 days, with 2-5 times higher colony numbers and larger colonies than the background, and increasing colony numbers with increasing concentrations of IPTG (IPTG activates the promoter on the plasmid, in this case for *hisA*; see methods). *∆ilvA/cysM* came up after 3 to 8 days, with medium background. Finally, the strongest new replacer was *∆metC/metB*, which came up after 2-3 days with no background. To understand better how these phenotypes relate to the gene similarity that enabled us to make the promiscuity predictions, we did a sequence alignment of each of the successful replacers (*hisA*, *cysM*, and *metB*) versus the gene closest to them in their trees that carried the function of the target. We found that, indeed, the strength of homology scaled with the strength of the observed phenotype, at least for these three instances (*metB* was highest, *cysM* middle, and *hisA* lowest -- compare alignments shown in supplement: Figure S4, Figure S5, and Figure S6). A more detailed experimental study should be done to confirm this trend generally.

### Determining targets to predict indirect replacers for

Although conditional *in vitro* lethality is a necessary condition for a gene to be a target in a multicopy suppression assay, many of Patrick’s targets are not conditionally lethal on M9 medium in our *in silico* model of *E. coli*. We checked these targets in a manually curated *E. coli* model, iAF1280, and similarly, many of them are not conditionally lethal *in silico* ^4^. In order to simplify our analysis, we therefore focused only on the subset of targets that were *in silico* lethal on M9 medium in the *E. coli* SEED metabolic model.

### Analysis of pathways using B_6_ as a cofactor

We predicted that thiG harbors a promiscuous functionality that can bypass the function of pdxB (see Figure 4 in the main text). This key function of pdxB that thiG replaces is production of pyridoxal 5’-phosphate (p5p), which is the active form of Vitamin B_6_, a cofactor used extensively across life. In our assays for validating our proposed active site of thiG for the secondary pdxB-replacing activity, it occurred to us that we might be able to alter the sensitivity of the assay by altering the cellular demand for Vitamin B_6_. A higher B_6_ demand would make p5p more essential, and thus would make it more critical for the cell to bypass the lost pdxB function, and thus increase the signal to noise ratio.

To determine how Vitamin B_6_ is used in *E. coli* and thus how we might alter demand for it, we extracted the list of *E. coli* enzymes that utilize B_6_ as a cofactor from the B6 Database: ^5^. We then classed those enzymes into pathways (see Supplementary Figure S10). A quick survey of the top B_6_ utilizing pathways reveals that amino acid pathways dominate the list, and since amino acids are large nitrogen consumers, we hypothesized that altered levels of nitrogen consumption might have some effect on the demand for p5p, and thus might influence the signal to noise ratio in our validation experiments for thiG secondary activity. Based on this, we chose the concentration of NH4Cl (the Nitrogen source in M9 medium), alongside the concentration of the plasmid inducer (IPTG), as variables to vary across the wells of a microplate in determining optimal growth conditions to maximize the strength of our signal in our thiG validation assay (see Figure 5a in the main text).

### Expanded analysis of *thiGmut* growth dynamics

As reported in Figure 5 of the main text, we ran a microwell plate experiment to determine the differences in growth between *ΔpdxB/pdxB*, *ΔpdxB/thiG*, *ΔpdxB/thiGmut*, and *ΔpdxB/empty*, which constitute the *ΔpdxB* strain containing a plasmid with: (1) a positive control (self-replacement), (2) the predicted indirect replacer *thiG*, (3) the *thiG* gene with its proposed promiscuous active site altered, or (4) an empty plasmid negative control.

We saw a clear distinction in that experiment between two groups: *ΔpdxB/pdxB* and *ΔpdxB/thiG* clustered together with robust growth, while *ΔpdxB/thiGmut* and *ΔpdxB/empty* clustered together with weak growth (see Figure 5). These results were reported at the 96 hour timepoint, which is consistent with the timing at which distinguishable growth could be seen in the original plate count assay (colonies of *ΔpdxB/thiG* appeared on minimal media plates after 3-4 days; see Supplementary Table S2).

We also used the increased resolution afforded by these microwell batch experiments over the original plate experiment to examine earlier timepoints. At 24 hours, no distinction was noticeable between *ΔpdxB/thiG*, *ΔpdxB/thiGmut*, or *ΔpdxB/empty*. However, at 48 hours, a clear distinction is seen between growth of *ΔpdxB/thiG* and *ΔpdxB/empty* (p=7.0e-3) and between *ΔpdxB/thiG* and *ΔpdxB/thiGmut* (p=3.0e-3), but not between *ΔpdxB/thiGmut* and *ΔpdxB/empty* (p=0.10) (all p-values are ranksum tests of OD600 values across IPTG concentrations at 3% NH4Cl from the microwell plates, as reported for the 96 hour timepoint in the main text; see Supplementary Figure S11a).

To confirm this trend, we performed a follow-up batch culture experiment in 30mL batches under constant agitation, set at the optimal growth conditions determined in the microwell assay (these were: 3% w/v NH4Cl and 10uM IPTG; we did between 5 and 8 replicates per condition; see Methods). These experiments were only carried out to 48 hours due to evaporation considerations. This experiment revealed a significant (p<0.05 in 1-sided t-test) difference between ΔpdxB/thiG and ΔpdxB/empty and between ΔpdxB/thiG and ΔpdxB/thiGmut at all tested timepoints after 20 hours, but no significant difference between ΔpdxB/empty and ΔpdxB/thiGmut at any tested timepoint (see Figure 5). This further supports the results from the microwell experiment, namely, that mutating the proposed active site for the secondary function of *thiG* removes its replacer activity for *pdxB* (see Supplementary Figure S11b).

## References

1. Patrick WM, Quandt EM, Swartzlander DB, Matsumura I. Multicopy suppression underpins metabolic evolvability. *Mol Biol Evol* **24**, 2716-2722 (2007).

2. Soo VW, Hanson-Manful P, Patrick WM. Artificial gene amplification reveals an abundance of promiscuous resistance determinants in Escherichia coli. *Proc Natl Acad Sci U S A* **108**, 1484-1489 (2011).

3. Kim J, Kershner JP, Novikov Y, Shoemaker RK, Copley SD. Three serendipitous pathways in E. coli can bypass a block in pyridoxal-5'-phosphate synthesis. *Mol Syst Biol* **6**, 436 (2010).

4. Feist AM*, et al.* A genome-scale metabolic reconstruction for Escherichia coli K-12 MG1655 that accounts for 1260 ORFs and thermodynamic information. *Mol Syst Biol* **3**, 121 (2007).

5. Percudani R, Peracchi A. The B6 database: a tool for the description and classification of vitamin B6-dependent enzymatic activities and of the corresponding protein families. *BMC Bioinformatics* **10**, 273 (2009).
