# Supplementary figures and images for: Systems-Wide Prediction of Enzyme Promiscuity Reveals a New Underground Alternative Route for Pyridoxal 5’-Phosphate Production in E. coli
Source: PLoS Comput Biol. 2016 Jan 28;12(1):e1004705. doi: 10.1371/journal.pcbi.1004705 (PMC4731195; doi:10.1371/journal.pcbi.1004705)

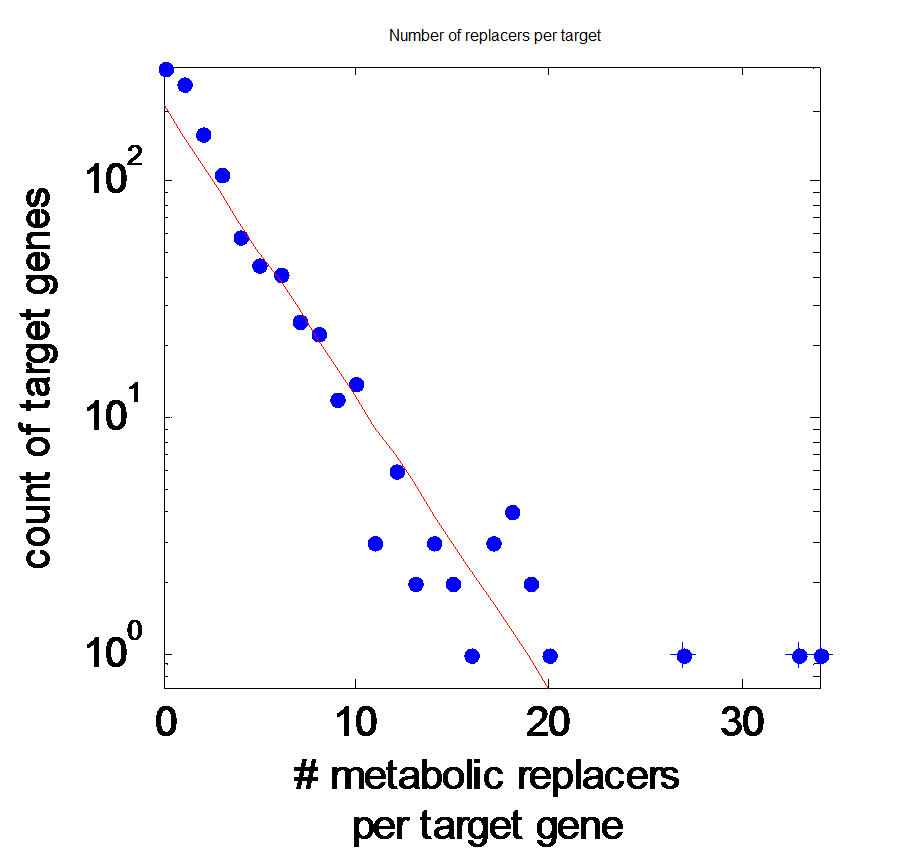

Supplement: S1 Fig — (TIF) [file pcbi.1004705.s002.tif]

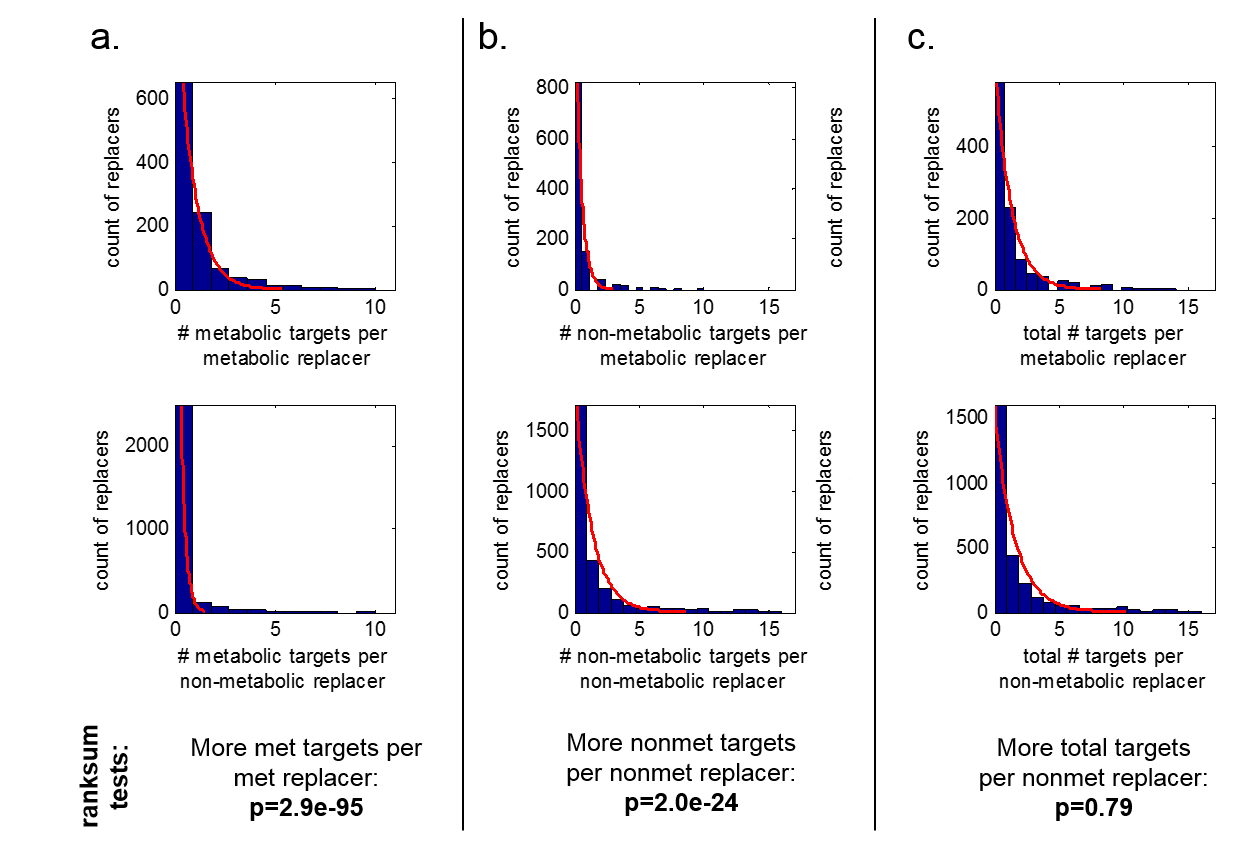

Supplement: S2 Fig — All target-replacer pairs predicted by our method were assessed for how often a metabolic target paired with a metabolic replacer, with a non-metabolic replacer, a non-metabolic target with a metabolic replacer, etc. We found that (A) Replacer genes with metabolic primary functions take more metabolic targets than do replacer genes with non-metabolic primary functions; (B) Replacer genes with non-metabolic primary functions take more non-metabolic targets than do replacer genes with metabolic primary functions; and (C) The number of targets replaced by replacer genes with non-metabolic vs. metabolic primary functions is the same. (TIF) [file pcbi.1004705.s003.tif]

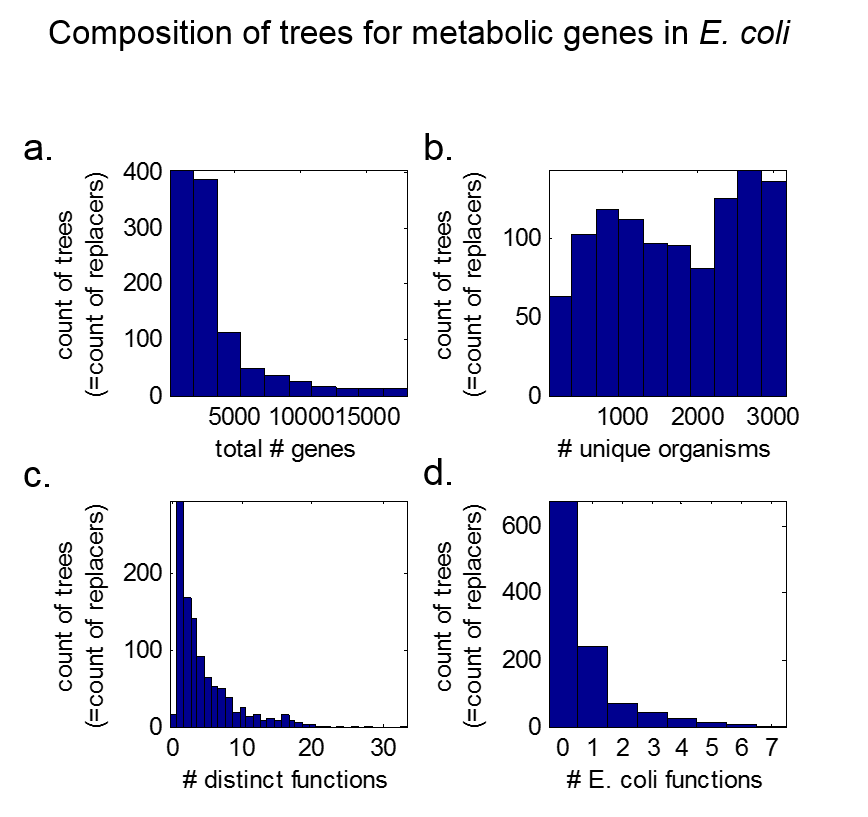

Supplement: S3 Fig — Histograms are shown of: (A) number of genes represented in each tree; (B) number of unique organisms represented in each tree; (C) number of distinct functions represented in each tree; and (D) number of E. coli metabolic functions present in each tree. (TIF) [file pcbi.1004705.s004.tif]

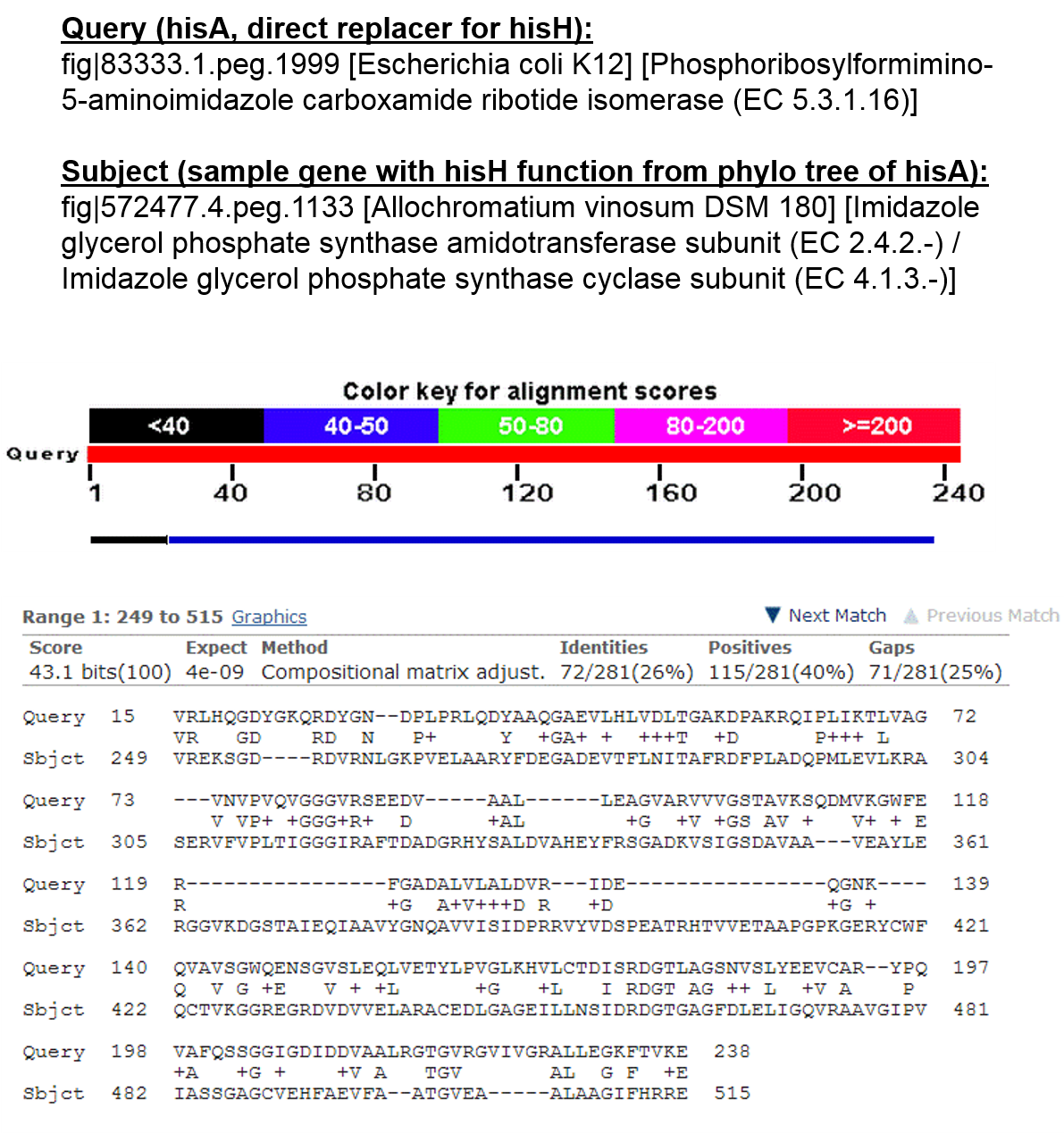

Supplement: S4 Fig — (TIF) [file pcbi.1004705.s005.tif]

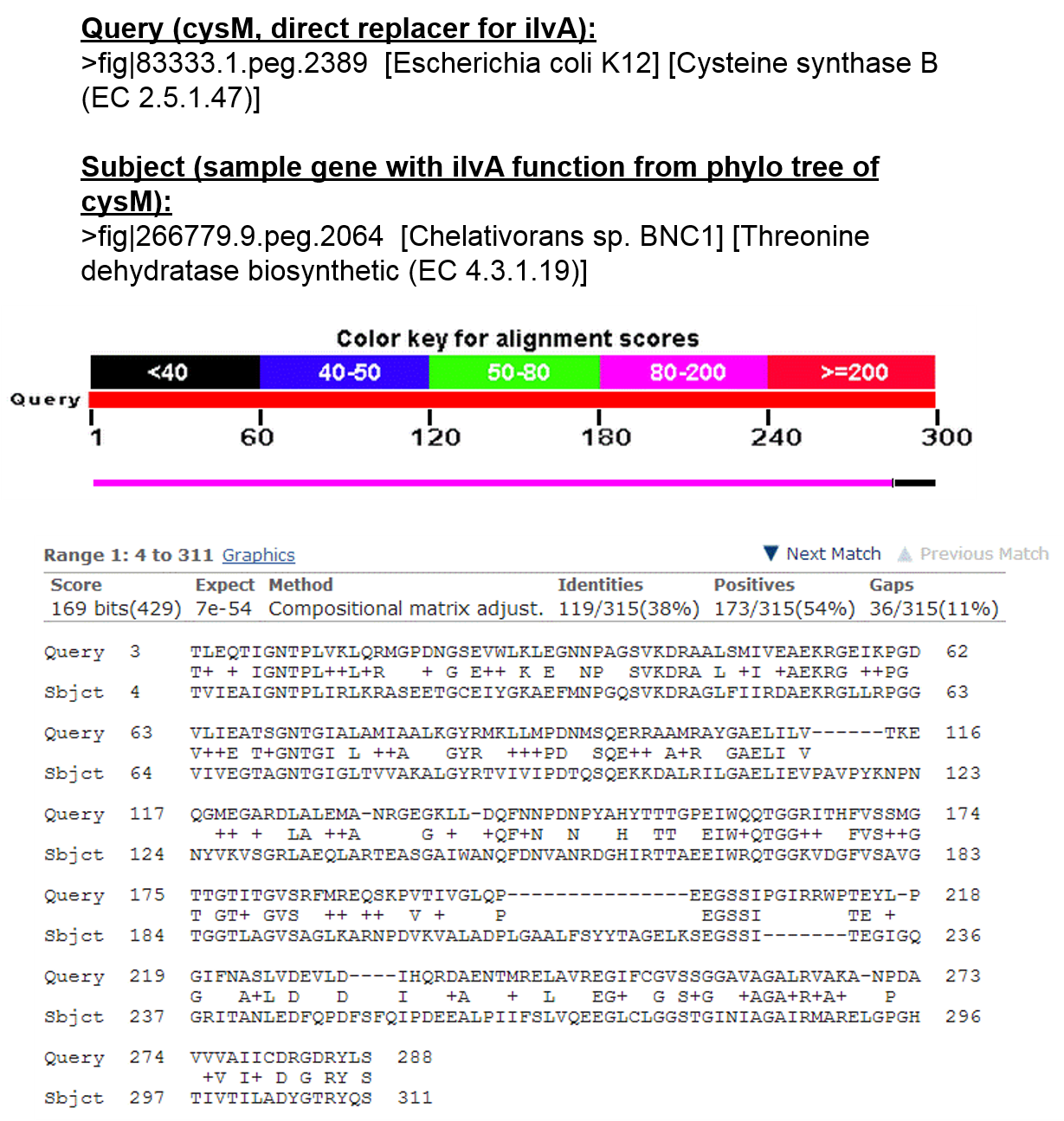

Supplement: S5 Fig — (TIF) [file pcbi.1004705.s006.tif]

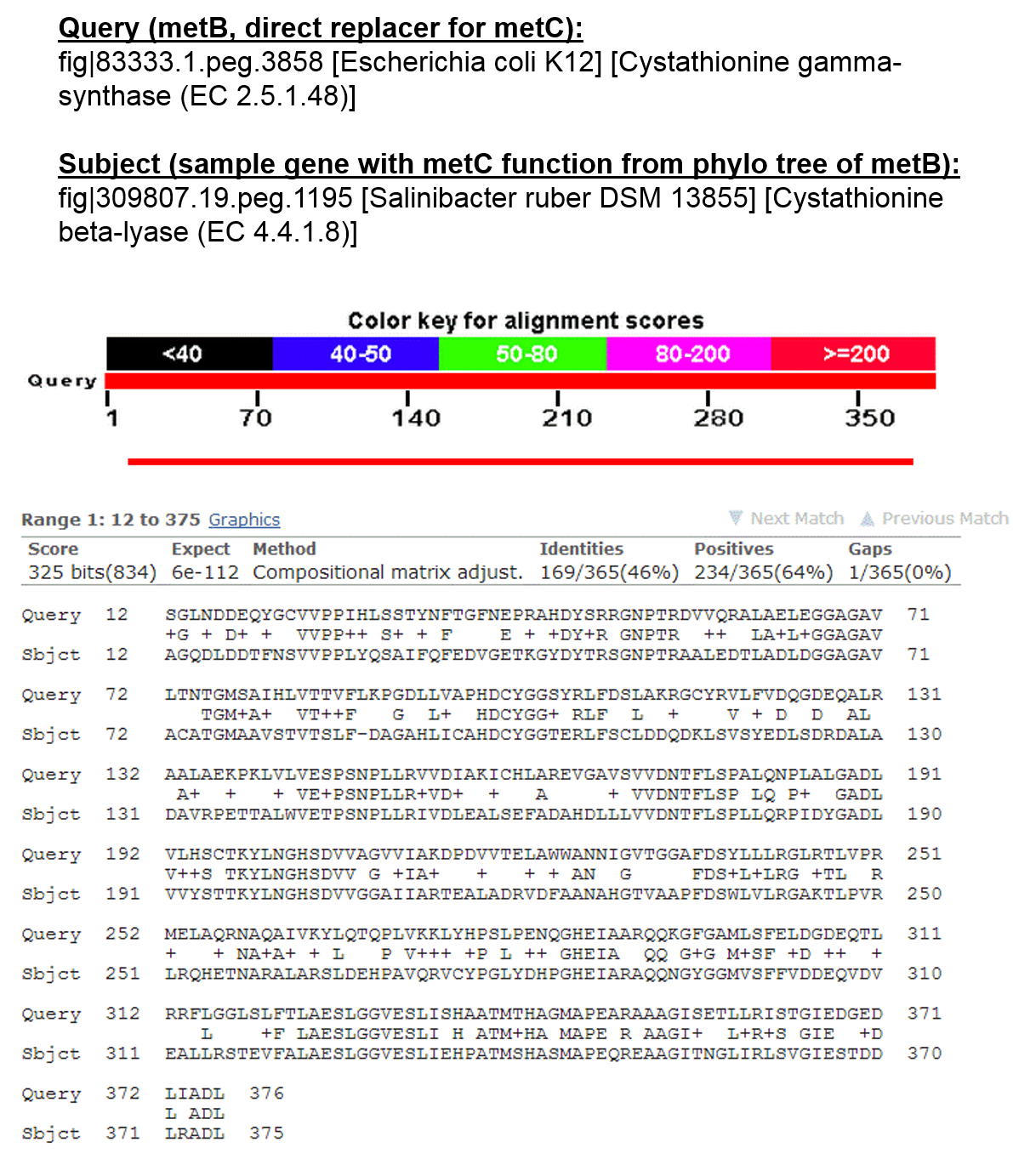

Supplement: S6 Fig — (TIF) [file pcbi.1004705.s007.tif]

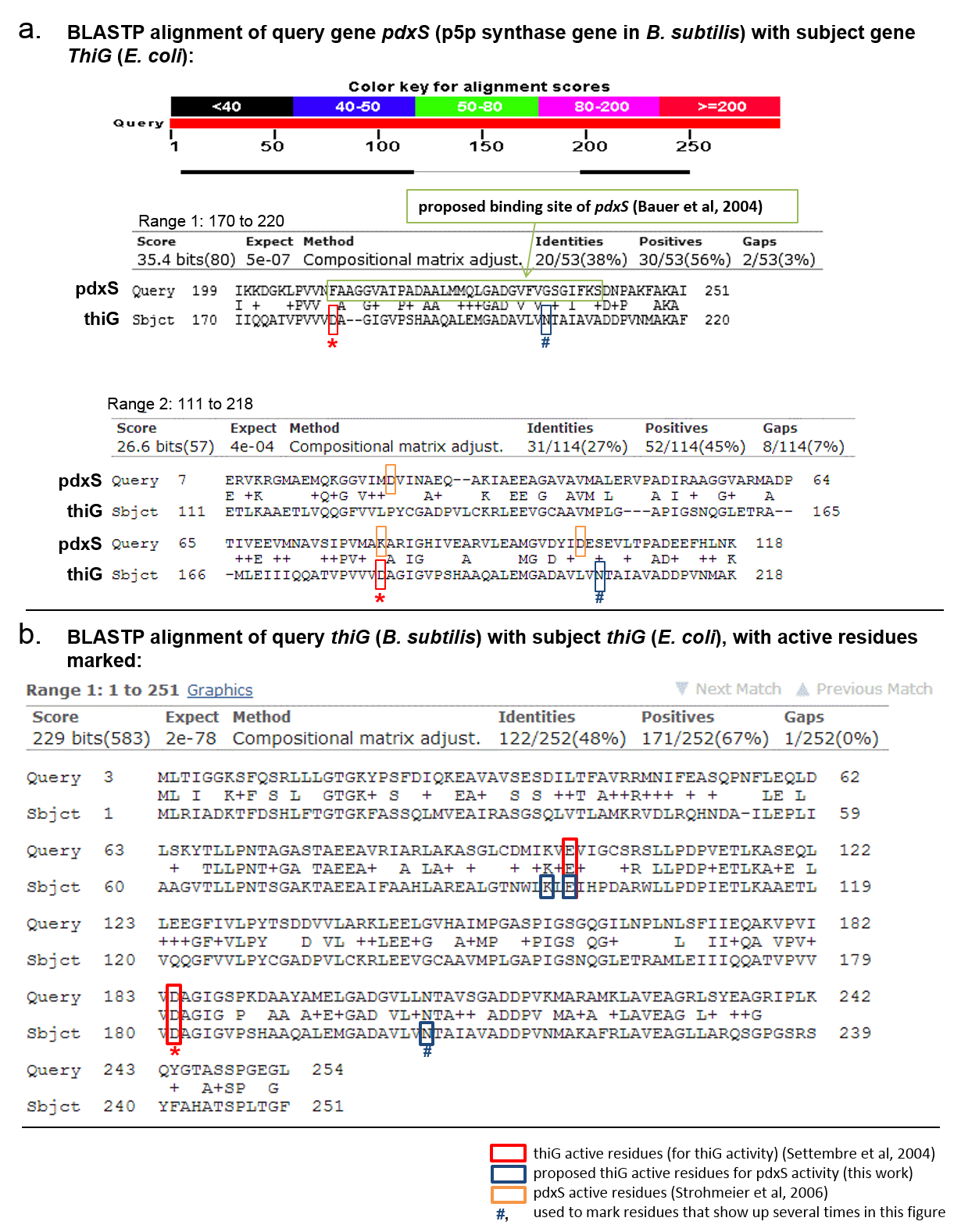

Supplement: S7 Fig — (A) E. coli thiG is aligned with pdxS, the gene whose function it putatively performs promiscuously. Key residues in both genes are marked, as per the key at the bottom. Two alignments are shown as they came up in BLASTP sequence alignment. (B) The active residues of thiG for its primary function are shown, along with putative active residues for the pdxS function. (TIF) [file pcbi.1004705.s008.tif]

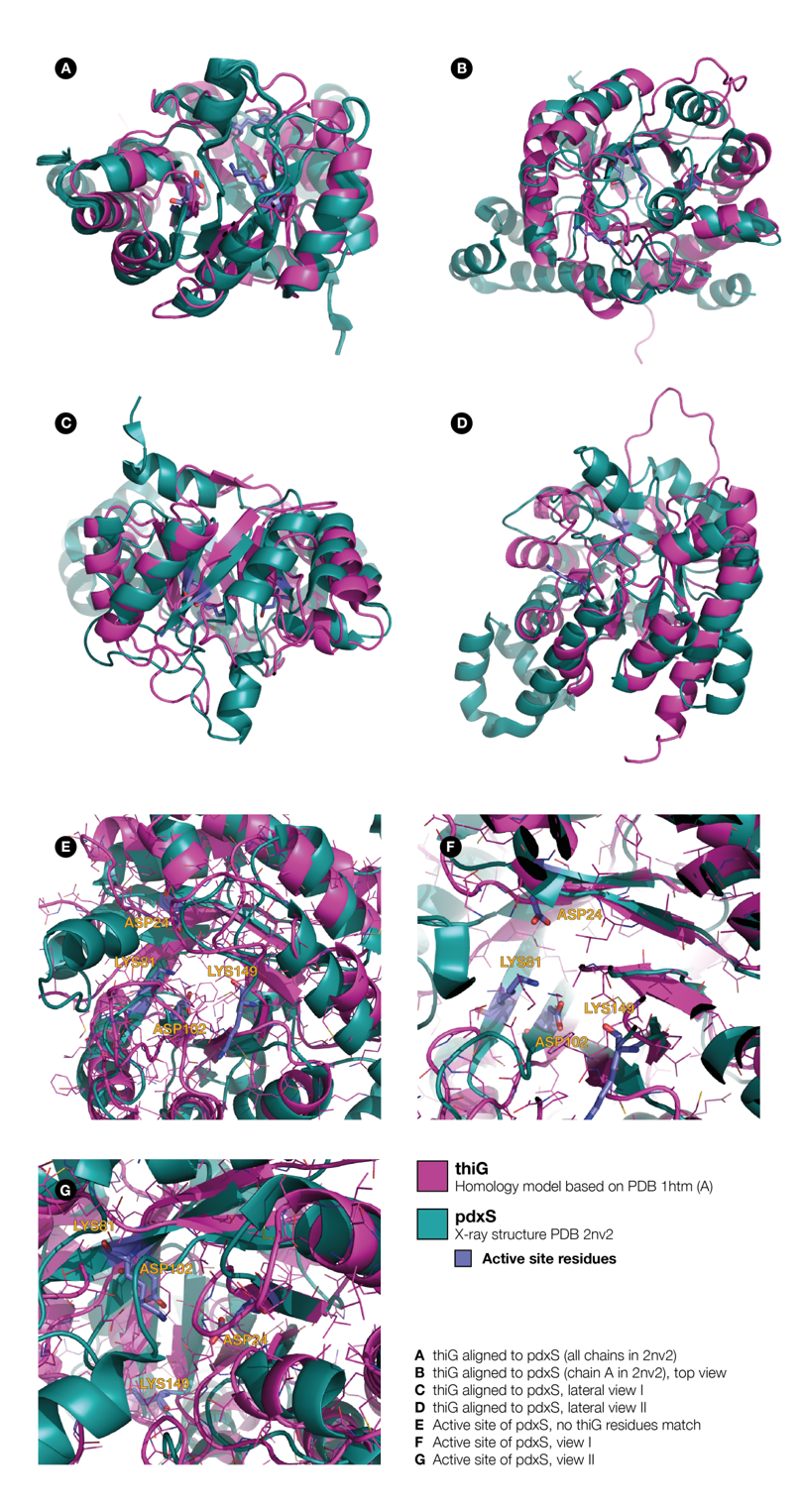

Supplement: S8 Fig — Multiple alignments are shown. (TIF) [file pcbi.1004705.s009.tif]

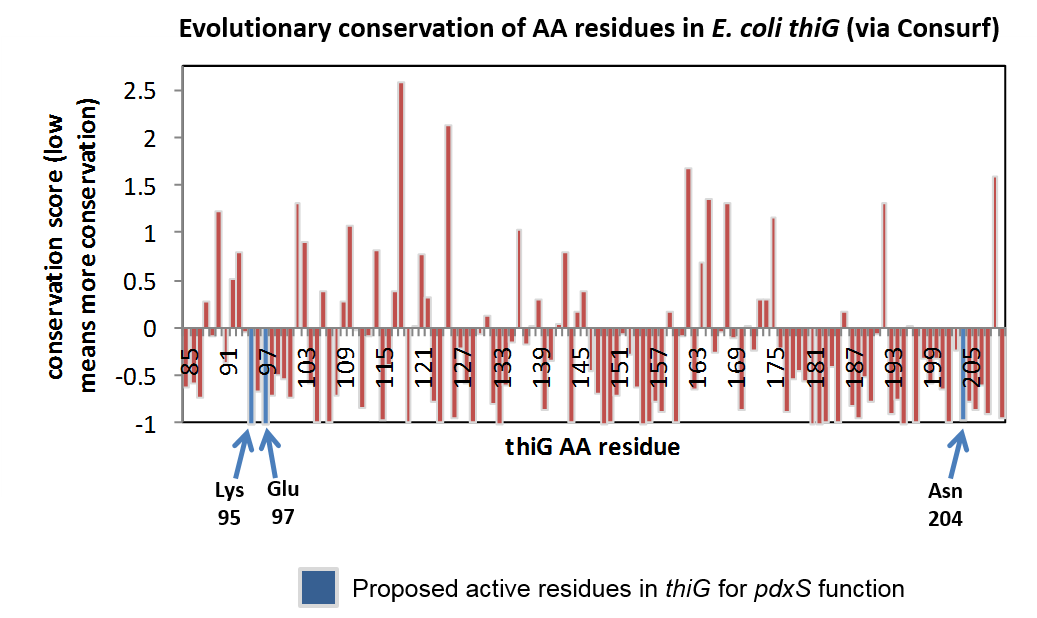

Supplement: S9 Fig — The residues in thiG that we predict can perform the pdxS function show a high degree of conservation, as shown in the plot. This was generated from the output of ConSurf, set with default settings except for a maximum cutoff of 70% homology in the sequences to be aligned (higher cutoffs led to less resolution in distinguishing how conserved the key residues are versus their neighbors). (TIF) [file pcbi.1004705.s010.tif]
