# Supplementary material for: Systems-Wide Prediction of Enzyme Promiscuity Reveals a New Underground Alternative Route for Pyridoxal 5’-Phosphate Production in E. coli
Source: PLoS Comput Biol. 2016 Jan 28;12(1):e1004705. doi: 10.1371/journal.pcbi.1004705 (PMC4731195; doi:10.1371/journal.pcbi.1004705)
Supplement: Supplementary file 3 [file pcbi.1004705.s011.docx]

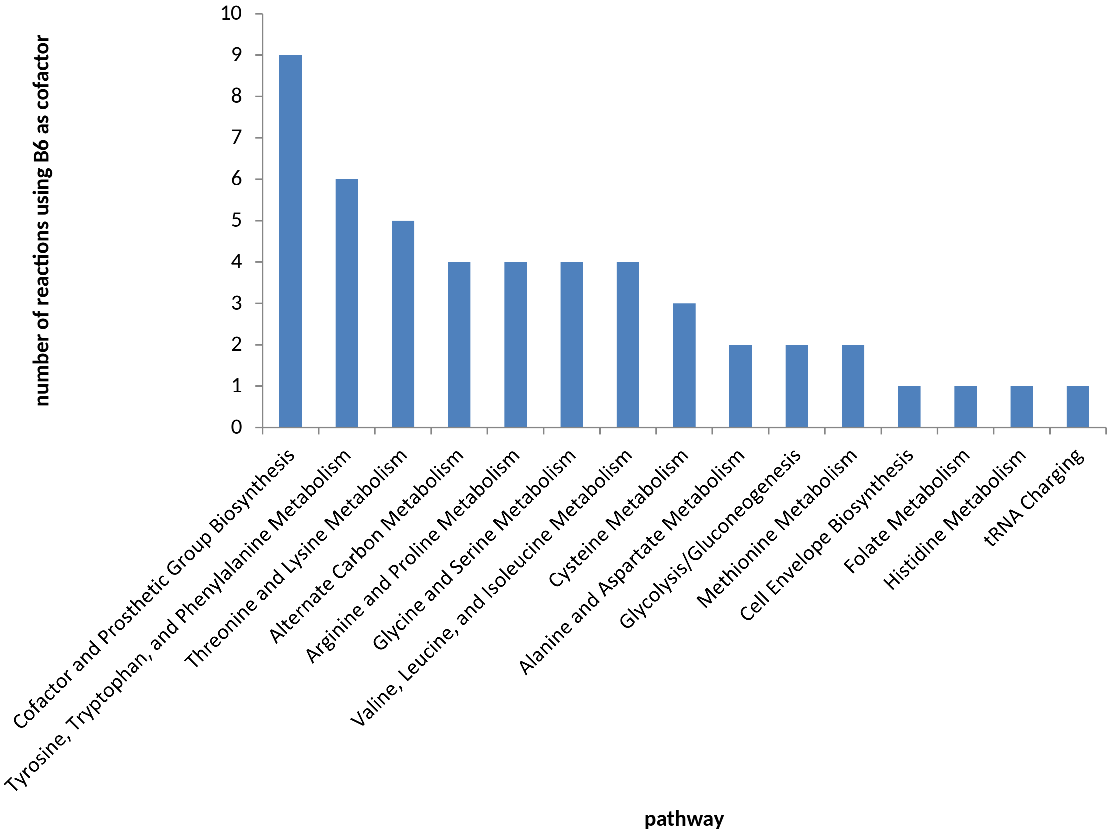
number of reactions using B6 as cofactor
10
9
8
7
6
5
4
3
2
1
0
Cofactor and Prosthetic Group Biosynthesis
Tyrosine, Tryptophan, and Phenylalanine Metabolism
Threonine and Lysine Metabolism
Alternate Carbon Metabolism
Arginine and Proline Metabolism
Glycine and Serine Metabolism
Valine, Leucine, and Isoleucine Metabolism
Cysteine Metabolism
Alanine and Aspartate Metabolism
Glycolysis/Gluconeogenesis
Methionine Metabolism
Cell Envelope Biosynthesis
Folate Metabolism
Histidine Metabolism
tRNA Charging
pathway
