# Supplementary material for: Systems-Wide Prediction of Enzyme Promiscuity Reveals a New Underground Alternative Route for Pyridoxal 5’-Phosphate Production in E. coli
Source: PLoS Comput Biol. 2016 Jan 28;12(1):e1004705. doi: 10.1371/journal.pcbi.1004705 (PMC4731195; doi:10.1371/journal.pcbi.1004705)
Supplement: Supplementary file 4 [file pcbi.1004705.s012.docx]

a.

Ranksum tests across IPTG concentrations
(at 4% NH4Cl in the microwell plate assay)
48 hour timepoint

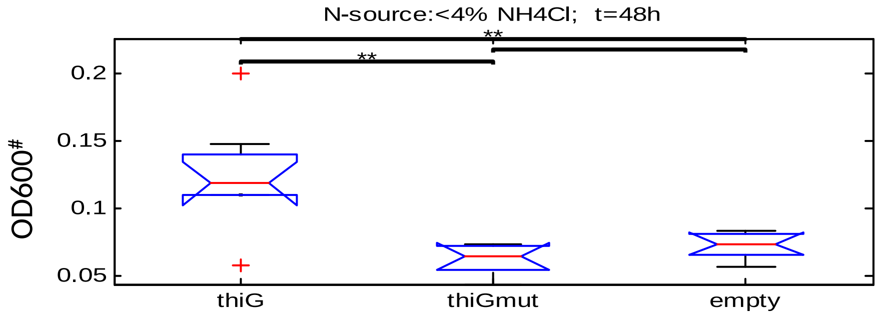

ΔpdxB/thiG   ΔpdxB/thiGmut   ΔpdxB/empty

b.

T-tests across 5-8 replicates in batch culture (4% NH4Cl, 10uM IPTG)
48 hour timepoint

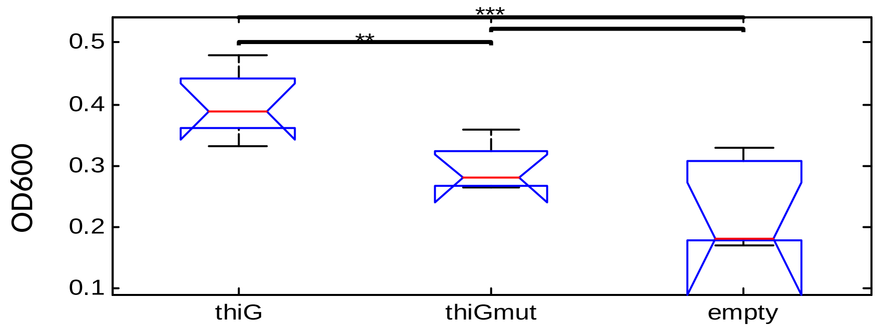

ΔpdxB/thiG   ΔpdxB/thiGmut   ΔpdxB/empty

<u>Key to p-values:</u>
* denotes p<=0.05
** denotes p<=1e-2
*** denotes p<=1e-3
